# Occupancy of the Zinc-binding Site by Transition Metals Decreases the Substrate Affinity of the Human Dopamine Transporter by an Allosteric Mechanism*[Fn FN2]

**DOI:** 10.1074/jbc.M116.760140

**Published:** 2017-01-17

**Authors:** Yang Li, Felix P. Mayer, Peter S. Hasenhuetl, Verena Burtscher, Klaus Schicker, Harald H. Sitte, Michael Freissmuth, Walter Sandtner

**Affiliations:** From the Institute of Pharmacology, Center of Physiology and Pharmacology, Medical University Vienna, Waehringerstrasse 13a, 1090 Vienna, Austria

**Keywords:** dopamine, electrophysiology, metal, monoamine transporter, neurotransmitter transport

## Abstract

The human dopamine transporter (DAT) has a tetrahedral Zn^2+^-binding site. Zn^2+^-binding sites are also recognized by other first-row transition metals. Excessive accumulation of manganese or of copper can lead to parkinsonism because of dopamine deficiency. Accordingly, we examined the effect of Mn^2+^, Co^2+^, Ni^2+^, and Cu^2+^ on transport-associated currents through DAT and DAT-H193K, a mutant with a disrupted Zn^2+^-binding site. All transition metals except Mn^2+^ modulated the transport cycle of wild-type DAT with affinities in the low micromolar range. In this concentration range, they were devoid of any action on DAT-H193K. The active transition metals reduced the affinity of DAT for dopamine. The affinity shift was most pronounced for Cu^2+^, followed by Ni^2+^ and Zn^2+^ (= Co^2+^). The extent of the affinity shift and the reciprocal effect of substrate on metal affinity accounted for the different modes of action: Ni^2+^ and Cu^2+^ uniformly stimulated and inhibited, respectively, the substrate-induced steady-state currents through DAT. In contrast, Zn^2+^ elicited biphasic effects on transport, *i.e.* stimulation at 1 μm and inhibition at 10 μm. A kinetic model that posited preferential binding of transition metal ions to the outward-facing apo state of DAT and a reciprocal interaction of dopamine and transition metals recapitulated all experimental findings. Allosteric activation of DAT via the Zn^2+^-binding site may be of interest to restore transport in loss-of-function mutants.

## Introduction

The physiological role of the dopamine transporter (DAT/SLC6A3)^3^ is to clear the extracellular space from previously released dopamine and to replenish vesicular stores (1). Accordingly, the transport capacity of DAT shapes the synaptic response. We recently showed that the endogenous ligand Zn^2+^ increases the turnover rate of DAT (2). This action of Zn^2+^ is presumably of physiological relevance: Zn^2+^ is accumulated in synaptic vesicles via a dedicated transporter (ZnT3/SLC30A3) and released as a co-transmitter (3).

Numerous proteins require Zn^2+^ for their activity; it has been estimated that about 10% of the proteins encoded by the human genome bind Zn^2+^ (4). Similarly, about 30% of all enzymes are thought to require Zn^2+^ for catalysis (5). In addition, structural Zn^2+^-binding sites are important for protein stability, *e.g.* in transcription factors harboring the eponymous zinc fingers (6). Zn^2+^-binding sites rely on the permutation of specific arrangements of cysteine, histidine, aspartate, and glutamate residues and, in catalytic Zn^2+^ sites, of water. The resulting affinities range from picomolar to micromolar (7). However, other first-row transition metal ions can also be trapped by these binding sites (5, 8). As an approximation, the stability of their interaction increases across the period to a maximum stability for complexes containing Cu^2+^, resulting in the Irving-Williams series: Mn^2+^ < Fe^2+^ < Co^2+^ < Ni^2+^ < Cu^2+^ > Zn^2+^ (9). It is thought that mismetallation of proteins is prevented by controlling the concentration of metal ions in the compartment in which the target protein operates (4, 7).

Mismetallation of DAT may be of toxicological relevance. It has long been known that occupational exposure to manganese can result in a syndrome resembling idiopathic Parkinson's disease (10, 11). Studies in primates indicate that manganese poisoning impairs dopamine release in the basal ganglia (12). Loss-of-function mutations in the manganese transporter SLC39A14 result in manganese overload and cause a syndrome of childhood dystonia/parkinsonism (13). This is reminiscent of inactivating mutations of DAT/SLC6A3 (14). Inactivating mutations in SLC30A10, another manganese extruder, also gives rise to parkinsonism (15). Similarly, Wilson's disease is caused by a deficiency in the copper transporter ATP7B, which results in copper accumulation in the basal ganglia and produces, *inter alia*, Parkinsonian symptoms (16). Before the link between dopamine deficiency and Parkinson's disease had been established, it was noted that dopamine excretion was augmented in patients suffering from Wilson's disease (17). Accordingly, in this study, we explored the effect of transition metals on human DAT. In our analysis, we relied on electrophysiological recordings because they provided the time resolution required to dissect the actions of the transition metals on individual steps of the transport cycle. We observed that individual metals differ in their ability to affect the transport cycle of DAT because they differed in the extent to which they were subject to a reciprocal modulation by substrate.

## Results

### 

#### 

##### Effects of Transition Metals on the Steady-state Current through DAT

**FIGURE 1.**
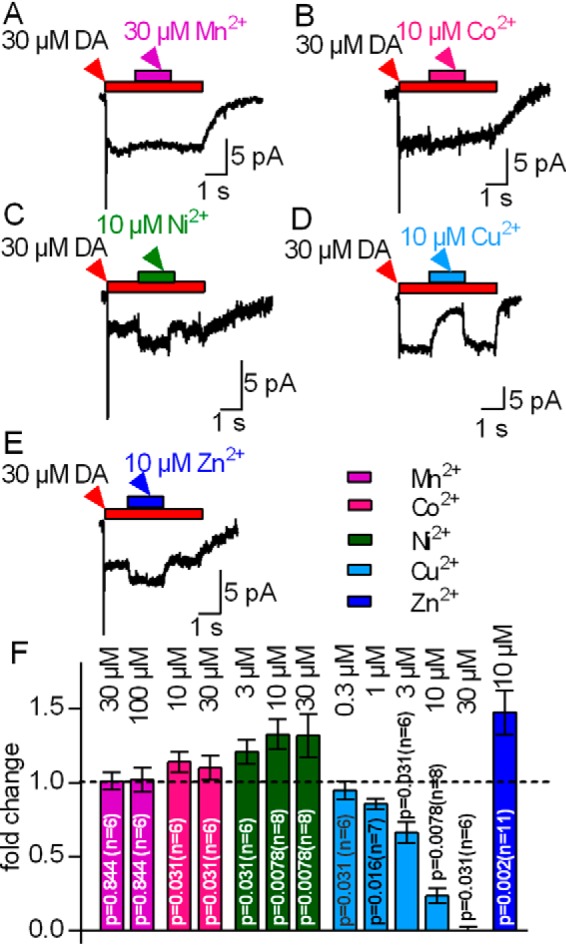
**Effects of first-row transition metals on the steady-state current amplitude (I_steady-state_) carried by hDAT.** HEK293 cells stably expressing hDAT were voltage-clamped to −60 mV. The cells were challenged with 30 μm dopamine (DA). After 2 s, the transition metal was co-applied for 3 s as indicated. *A*, co-application of 30 μm Mn^2+^ was ineffective. *B*, *C*, and *E*, co-application of 10 μm Co^2+^, Ni^2+^, or Zn^2+^ stimulated the current. *D*, 10 μm Cu^2+^ led to inhibition. In all instances, the steady-state current recovered to its initial amplitude upon metal removal. *F*, the steady-state current upon co-application of the indicated transition metals was related to that seen prior to metal application (-fold change = I_steady-state_ _after application_ / I_steady-state before application_) to normalize for differences in current sizes in individual cells. Data represent mean ± S.D. The respective -fold changes were tested against the hypothetical value 1 (= no change, indicated as a *dotted line* in the bar graph) using Wilcoxon matched pairs signed-rank test.

When challenged with substrate, monoamine transporters of the SLC6 family produce two types of currents that can be recorded in the whole-cell patch clamp configuration: an initial capacitive peak current that reflects the binding of substrate and co-substrate ions to the transporter and their movement in the electric field of the membrane (18, 19) and a sustained, steady-state current that reflects movement of the transporter through the transport cycle (18). These two components can be seen when dopamine is applied to a voltage-clamped HEK293 cell expressing DAT in the whole-cell patch clamp configuration (*cf.* Fig. 1, *A–E*). Zn^2+^ accelerates the return step, *i.e.* the transition of the empty inward-facing to the outward-facing state of the transporter (2). The resulting acceleration of the transport cycle resulted in an increase in the steady-state current when Zn^2+^ was applied while the cell was continuously superfused with dopamine (Fig. 1*E*). We employed Zn^2+^ at 10 μm because this was shown previously to be a saturating concentration (20–23). We compared the action of the transition metals Mn^2+^, Co^2+^, Ni^2+^, and Cu^2+^ (Fig. 1, *A–D*) with that of Zn^2+^ (Fig. 1*E*). We found Mn^2+^ to be ineffective at concentrations between 1 and 100 μm. In Fig. 1, *A* and *F*, we show the absence of any effect at 30 and 100 μm.

**FIGURE 2.**
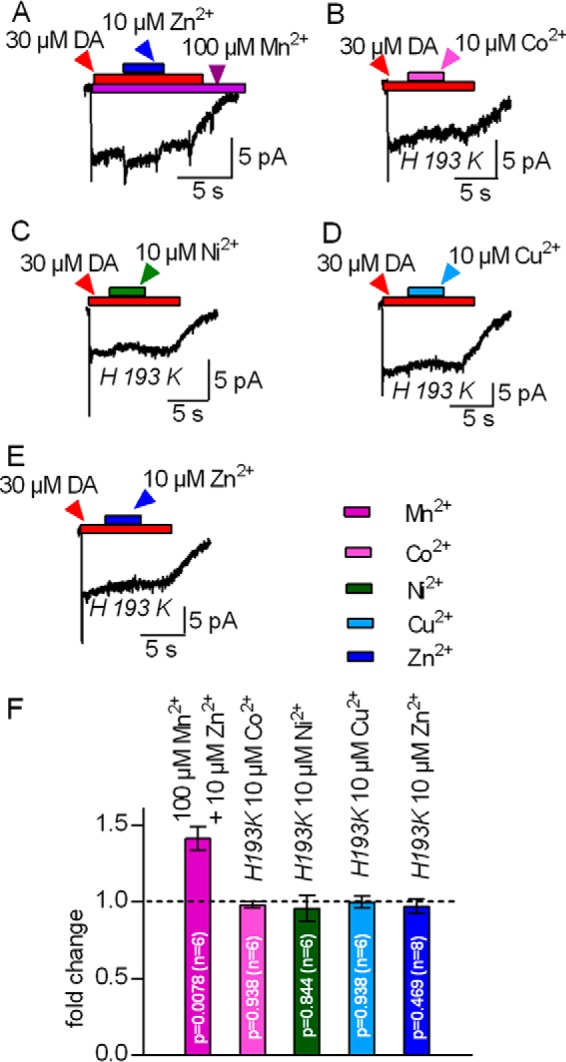
**Interaction of Co^2+^, Ni^2+^, and Cu^2+^ but not Mn^2+^ with the endogenous Zn^2+^-binding site of DAT.** *A–E*, HEK293 cells stably expressing hDAT (*A*) or transiently transfected with the mutant hDAT-H193K (*B–E*) were clamped to −60 mV. Currents were evoked by 30 μm dopamine. *A*, representative trace of a current recorded in the continuous presence of 100 μm Mn^2+^. Co-application of 10 μm Zn^2+^ increased the current amplitude. *B–E*, representative traces of currents carried by DAT-H193K. Co-application of 10 μm Co^2+^, Ni^2+^, Cu^2+^, or Zn^2+^ did not affect the current. *F*, summary of *A–E*. The steady-state current upon co-application of the indicated transition metals was related to that seen prior to metal application (-fold change); Zn^2+^ increased the current carried by hDAT in the continuous presence of 100 μm Mn^2+^. Currents by hDAT-H193K were unchanged upon co-application of 10 μm Co^2+^, Ni^2+^, Cu^2+^, or Zn^2+^, respectively. Data represent mean ± S.D. The respective -fold changes were tested against the hypothetical value 1 (= no change, indicated as a *dotted line* in the bar graph) using Wilcoxon matched pairs signed-rank test.

In contrast, application of 10 μm Co^2+^ (Fig. 1*B*) led to a small but significant increase in the steady-state current amplitude by about 10%. The current did not increase further in the presence of 30 μm Co^2+^ (Fig. 1*F*). This weak stimulatory effect of Co^2+^ precluded further analysis. Application of 10 μm Ni^2+^ resulted in a robust increase in the steady-state current, which relaxed to its initial amplitude upon removal of Ni^2+^ (Fig. 1*C*). We also tested 3 and 30 μm Ni^2+^ (summarized in Fig. 1*F*). The effect elicited by 30 μm Ni^2+^ was essentially comparable with that caused by 10 μm Ni^2+^, indicating that, at saturating levels of Ni^2+^, the steady-state currents were about 1.3-fold larger than in the absence of any metal. Contrary to Co^2+^ and Ni^2+^, Cu^2+^ reversibly inhibited the steady-state current through DAT (Fig. 1*D*) with an IC_50_ of 4.4 μm (2.3–8.2 μm, 95% confidence interval). These observations suggest that all tested transition metals except Mn^2+^ either stimulate or inhibit the transport cycle of DAT. Two explanations can account for the inability of Mn^2+^ to modulate the activity of DAT: Mn^2+^ binds but neither stimulates nor inhibits the transport cycle, or the affinity of Mn^2+^ for the Zn^2+^-binding site of DAT is too low. We distinguished between these two possibilities by first concomitantly applying 30 μm dopamine and 100 μm Mn^2+^ to elicit the steady-state current through DAT. If Mn^2+^ had occupied the Zn^2+^-binding site of DAT, then it should preclude the increase in steady-state current resulting from the subsequent application of Zn^2+^. This, however, was not the case. As can be seen in Fig. 2*A*, wash-in of Zn^2+^ induced a robust stimulation of the current that was similar to that seen in the absence of Mn^2+^ (*cf.* Fig. 2*F*, *first* and *fifth columns*).

We also verified that both the stimulatory action of Co^2+^ and Ni^2+^ and the inhibitory action of Cu^2+^ required the Zn^2+^-binding site of DAT by examining their action on steady-state currents through DAT-H193K. In this mutant, the Zn^2+^-coordinating histidine is replaced by lysine, the residue found at the equivalent position of the norepinephrine transporter. The norepinephrine transporter is the closest relative of DAT but is insensitive to Zn^2+^. Accordingly, the mutation of His^193^ to Lys eliminates high-affinity Zn^2+^ binding to DAT (20, 21). It is evident from the current traces shown in Fig. 2, *B–E*, and the summary shown in Fig. 1*F* that both the stimulation of the steady-state current by Co^2+^, Ni^2+^, and Zn^2+^ and its inhibition by Cu^2+^ were abrogated in DAT-H193K. This comparison is based on recordings done in a stable cell line and transiently transfected cells for wild-type DAT (Fig. 1) and the DAT-H193K mutant (Fig. 2), respectively. We also recorded currents through wild-type DAT in transiently transfected HEK293 cells. These experiments recapitulated the data shown in Fig. 1, *i.e.* stimulation of substrate-induced currents by Co^2+^, Ni^2+^, and Zn^2+^, their inhibition by Cu^2+^, and the absence of any effect in the presence of Mn^2+^ (data not shown). Hence, we rule out that differences between transiently and stably transfected cells can account for the distinct effects of transition metals on wild-type and DAT-H913K.

##### Transition Metal-induced Shifts of EC_50_ for Substrate-induced Steady-state Currents through hDAT

**FIGURE 3.**
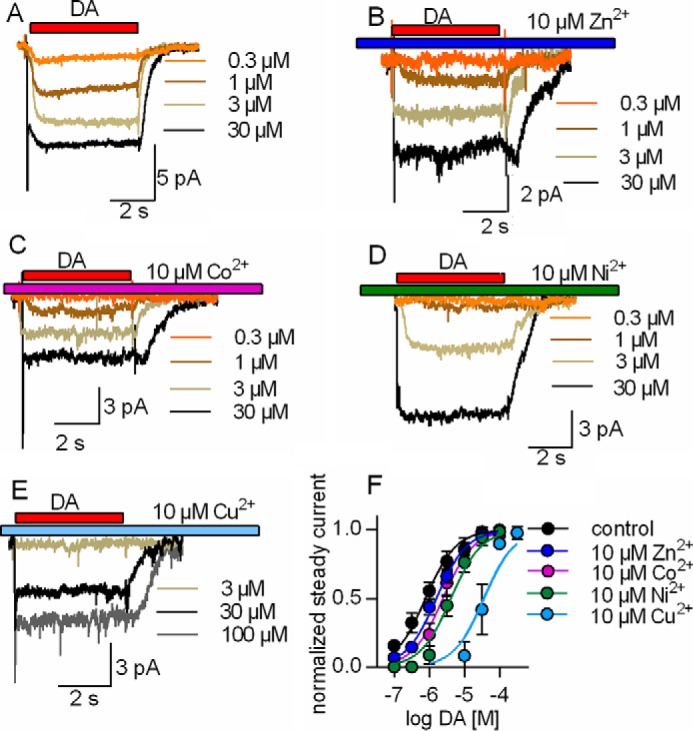
**Rightward shift by Zn^2+^, Co^2+^, Ni^2+^, and Cu^2+^ in the EC_50_ of dopamine in eliciting the steady-state current through DAT.** HEK293 cells stably expressing hDAT were clamped to −60 mV. Currents through DAT were evoked by 5-s applications of increasing dopamine concentrations (0.3–100 μm) in the absence and presence of 10 μm Zn^2+^, Co^2+^, Ni^2+^, and Cu^2+^. *A–E*, representative traces of currents through hDAT evoked by increasing dopamine concentrations in the absence (*A*) or presence of 10 μm Zn^2+^, Co^2+^, Ni^2+^, and Cu^2+^, respectively (*B–E*). *F*, the concentration-response curve of the steady-state current was shifted to higher dopamine concentrations in the presence of the metals. The amplitudes of the steady-state current were normalized to the current at saturation. The EC_50_ values were estimated by a fit to a binding hyperbola. EC_50 control_ = 0.8 μm (0.6–1, *n* = 9), EC_50 Zn2+_ = 1.6 μm (1.2–2.2, *n* = 8), EC_50 Co2+_ = 2.4 μm (2.1–2.8, *n* = 8), EC_50 Ni2+_ = 4.3 μm (3.5–4.7, *n* = 6), and EC_50 Cu2+_ = 36.5 μm (17.1–72.2, *n* = 6). Data represent mean and 95% confidence interval (in parentheses).

The substrate-induced steady-state current reflects cycling of DAT through the forward transport mode (18). Dopamine increased the steady-state current through DAT in a concentration-dependent manner (Fig. 3*A*). This concentration-response curve was shifted by all transition metals; representative original traces are shown in Fig. 3, *B–E*, for dopamine-induced steady-state currents in the presence of 10 μm Zn^2+^, Co^2+^, Ni^2+^, and Cu^2+^, respectively. The pertinent analysis for all transition metals is summarized in Fig. 3*E*; the concentration-response curve was adequately described by a saturation hyperbola. More importantly, all transition metals reduced the apparent affinity of dopamine, but the magnitude of the shift differed. In the absence of any metal, the EC_50_ for the currents induced by dopamine was 0.8 μm (0.6–1 μm, 95% confidence interval) and increased to EC_50 Zn2+_ = 1.6 μm (1.2–2.2 μm), EC_50 Co2+_ = 2.4 μm (2.1–2.8 μm), EC_50 Ni2+_ = 4.3 μm (3.5–4.7 μm), and EC_50 Cu2+_ = 36.5 μm (17.1–72.2 μm) in the presence of Zn^2+^, Co^2+^, Ni^2+^, and Cu^2+^, respectively.

##### Sole Activation of DAT by Ni^2+^

**FIGURE 4.**
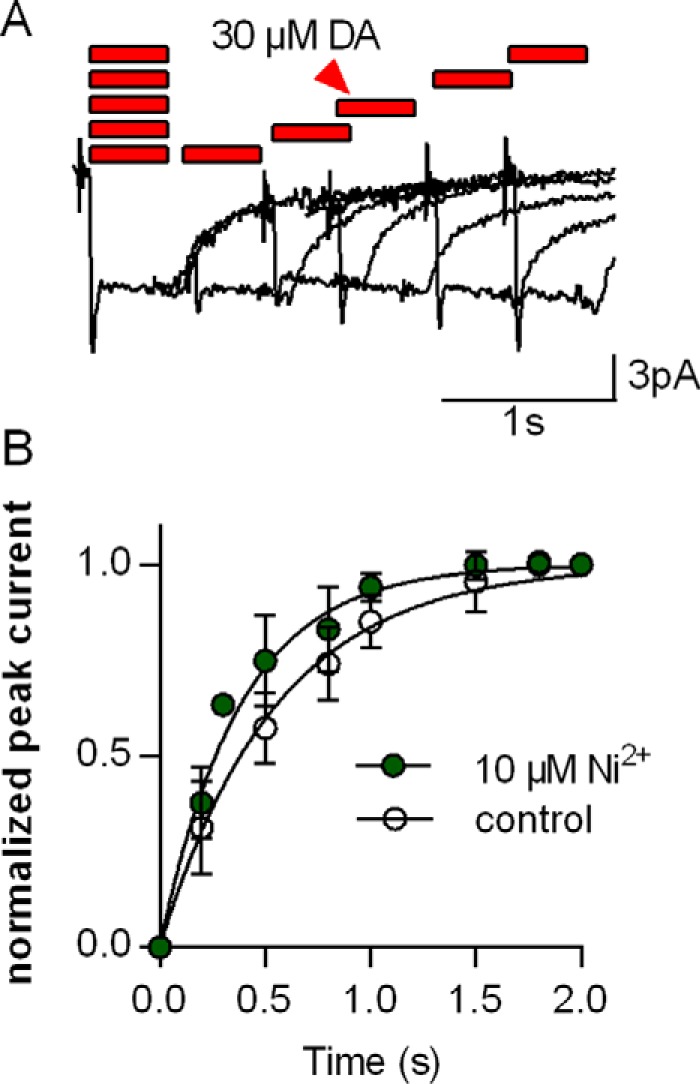
**Ni^2+^-induced increase in the turnover rate of DAT.** HEK293 cells stably expressing hDAT were voltage-clamped to −60 mV. *A*, protocol to measure the turnover rate of DAT. 30 μm dopamine was applied to the cell for 0.5 s. This was followed by a second pulse (30 μm dopamine for 0.5 s) applied after different wash intervals (0.1, 0.2, 0.5, 0.8, 1, 1.5, and 2 s). The peak current recovered over time and reached its full amplitude after prolonged dopamine-free intervals. *B*, the peak current recovers faster in the presence of 10 μm Ni^2+^ (*green circle*) in comparison with the control. The peak currents were normalized to the respective largest peak current from the same cell. The time course of the peak current recovery was fit to a monoexponential function (*black lines*, R^2^ = 0.96 and 0.96). The time constants estimated by the fits were as follows: control, 0.55 s (0.51–0.60, *n* = 6); Ni^2+^, 0.38 s (0.35–0.42, *n* = 6). Data represent mean and 95% confidence interval (in parentheses).

Taken together, the data summarized in Figs. 1–3 indicate that transition metals induced distinct effects on the transport cycle of DAT despite their binding to the same site: Cu^2+^ was uniformly inhibitory. The action of Zn^2+^ depends on the substrate concentration and the internal Na^+^ concentration (see below and Refs. 2, 22–24). Accordingly, we selected Ni^2+^ as an additional representative of the transition metals to understand their action on DAT because, contrary to Co^2+^, Ni^2+^ elicited a robust enhancement of the steady-state current. We recently demonstrated that current stimulation by Zn^2+^ is caused by an enhancement of the turnover rate of DAT (2). We verified that this was also true for Ni^2+^ by examining the turnover rate; the pertinent protocol and representative current traces are shown in Fig. 4. The approach relies on the paired application of substrate (*i.e.* dopamine) pulses. The first application elicits a robust peak current that corresponds to the conformational transition associated with substrate binding and drives DAT into the transport cycle, which gives rise to the sustained steady-state current. This steady-state current decays when substrate is withheld. During this decay, only a fraction of the transporters are available for binding of dopamine because they have not yet reached the outward-facing conformation. Full recovery of the peak current is achieved only after all transporters have completed the transport cycle. Hence, the rate of peak current recovery is a measure of the turnover rate. The time-dependent recovery of the peak current is evident from the original traces shown in Fig. 4*A*. The shorter the interval between the first and the second substrate pulse, the smaller a peak current was elicited by the second dopamine pulse. Full recovery was accomplished within 2 s with a time course that was adequately described by a monoexponential rise (Fig. 4*B*, *open symbols*. In the presence of Ni^2+^, the peak current recovered at a faster rate (Fig. 4*B*, *closed symbols*). Thus, similar to Zn^2+^, Ni^2+^ significantly accelerated the transport cycle of hDAT (*p* < 0.0001, F test).

**FIGURE 5.**
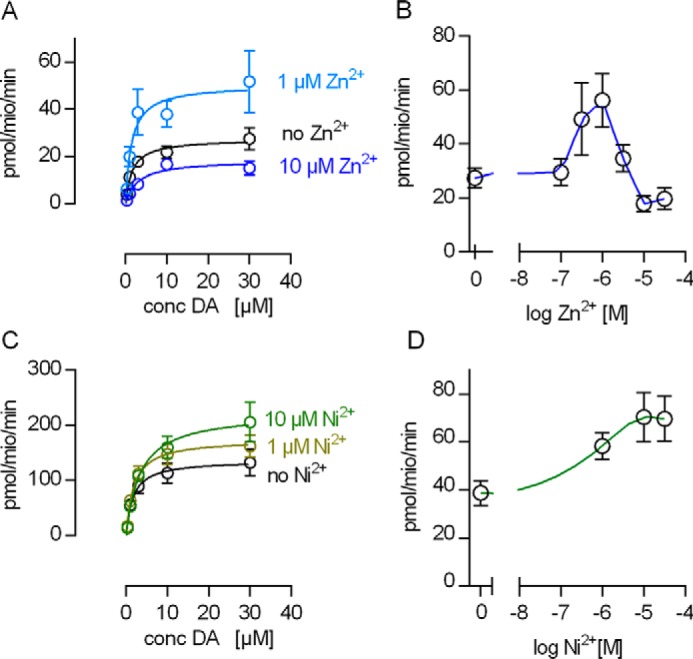
**Ni^2+^-mediated stimulation of substrate uptake by hDAT.** *A* and *C*, [^3^H]dopamine (DA) uptake in the absence and in the presence of 1 and 10 μm Zn^2+^ (*A*) or Ni^2+^ (*C*), respectively. Each data point in *A* and *C* is the mean of five independent experiments. The data were fit to the Michaelis-Menten equation, and the estimated parameters were as follows (*K_m_* in micromolar, *V*_max_ in picomoles per million per minute). *A*, control, *K_m_* = 1.6 (0.6–2.7, *V*_max_ = 27.3 (22.6–31.9); 1 μm Zn^2+^, *K_m_* = 1.5 (0.5–3.0), *V*_max_ = 50.3 (35.8–64.0); 10 μm Zn^2+^, *K_m_* = 2.9 (0.9–4.9), *V*_max_ = 18.3 (14.0–21.0). *C*, control, *K_m_* = 1.6 (0.3–3.0), *V*_max_ = 136 (107.5–164.7); 1 μm Ni^2+^, *K_m_* = 1.8 (0.7–2.9), *V*_max_ = 174.3 (144.0–204.0); 10 μm Ni^2+^, *K_m_* = 3.4 (1.4–5.3), *V*_max_ = 223.2 (180.9–265.0). Data represent mean and 95% confidence interval (in parentheses). The estimated *V*_max_ values were significantly different between 0, 1, and 10 μm Zn^2+^ (*p* < 0.0008, F-test) and between 0, 1, and 10 μm Ni^2+^ (*p* < 0.0024, F-test). *B* and *D*, *V*_max_ (= [^3^H]dopamine uptake at a final concentration of 30 μm dopamine) as a function of the applied Zn^2+^ or Ni^2+^ concentration. The *blue line* in *B* and the *green line* in *D* show spline fits through the data points. The *error bars* indicate standard deviation (*n* = 6).

This analysis of currents through DAT is consistent with the interpretation that Ni^2+^ acts as a stimulator of substrate uptake. We verified this conjecture by comparing cellular uptake of dopamine by hDAT in the absence and presence of 1 and 10 μm Zn^2+^ (Fig. 5*A*) and Ni^2+^ (Fig. 5*C*). Uptake of [^3^H]dopamine uptake was stimulated at both Ni^2+^ concentrations. In contrast, [^3^H]dopamine uptake was stimulated by 1 μm Zn^2+^ but inhibited by 10 μm Zn^2+^. This biphasic action of Zn^2+^ on substrate uptake in HEK293 cells has not been reported previously (20, 21). However, in earlier studies, the action of Zn^2+^ on substrate uptake by hDAT was explored at substrate concentrations below the *K_m_* of the transporter. Thus, we surmise that the stimulation at low Zn^2+^ concentrations escaped detection in studies (20, 21) that examined the action of Zn^2+^ at non-saturating substrate concentrations. We explored the biphasic effect of Zn^2+^ in the presence of saturating substrate concentrations (30 μm dopamine) in the absence and presence of 0.1, 1, 3, 10, and 30 μm Zn^2+^ (Fig. 5*B*). Under these conditions, the stimulation of maximal transport velocity peaks at about 1 μm Zn^2+^. We also explored the effect of Ni^2+^ at saturating substrate concentrations (30 μm dopamine) in the absence and presence of 1, 10, and 30 μm Ni^2+^. We found substrate uptake stimulated for Ni^2+^ concentrations up to 30 μm (Fig. 5*D*). At concentrations exceeding 30 μm, Ni^2+^ causes a nonspecific inhibition of transport, *i.e.* this inhibition is also seen in DAT-H193K (data not shown). Accordingly, it was not possible to examine how higher concentrations of Ni^2+^ affected substrate transport via the transition metal ion-binding site.

##### Stimulation of Currents through DAT by Ni^2+^ at High [Na^+^]_i_

**FIGURE 6.**
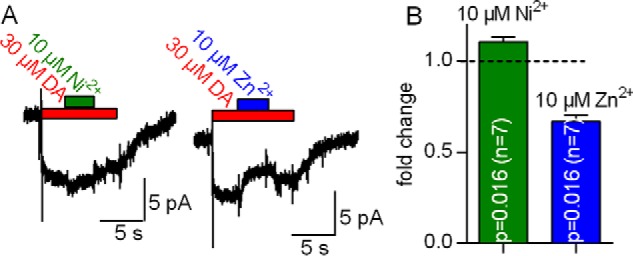
**Inhibition by Zn^2+^ but not by Ni^2+^ of steady-state currents through DAT in the presence of 25 mm [Na^+^]*_i_*.** *A*, a HEK293 cell stably expressing hDAT was clamped to −60 mV with [Na^+^]*_i_* set to 25 mm. The cell was challenged with 30 μm dopamine to elicit a current. Although co-application of 10 μm Ni^2+^ led to a small increase in the current amplitude, co-application of 10 μm Zn^2+^ decreased it. *B*, -fold change of the steady-state current at 25 mm [Na^+^]*_i_* in the presence of 10 μm Ni^2+^ (*green column*) and 10 μm Zn^2+^ (*blue column*). Data represent mean ± S.D. The respective -fold changes were tested against the hypothetical value 1 (= no change, indicated as a *dotted line* in the bar graph) using Wilcoxon matched pairs signed-rank test.

The action of Zn^2+^ depends on the intracellular sodium concentration. At low [Na^+^]*_i_*, Zn^2+^ acts as an activator of dopamine transport, but it is inhibitory when [Na^+^]*_i_* is high. This is the case in HEK293 cells overexpressing DAT (2, 22–24). Accordingly, the finding that Ni^2+^ increased substrate uptake into HEK293 cells suggested that Ni^2+^ can also stimulate DAT at high [Na^+^]*_i_*. We tested this conjecture by comparing the action of 10 μm Zn^2+^ and 10 μm Ni^2+^ on currents by DAT in the presence of 25 mm Na^+^_i_. Fig. 6*A* shows representative currents through hDAT that were induced by the rapid application of 30 μm dopamine followed by the concomitant superfusion with 10 μm Ni^2+^ (Fig. 6*A*, *left panel*) or 10 μm Ni^2+^ (Fig. 6*A*, *right panel*) for 5 s. It is evident from these recordings that, in the presence of 25 mm [Na^+^]*_i_*, Zn^2+^ inhibits the steady-state current whereas Ni^2+^ does not. Although we failed to detect current inhibition in the presence of 10 μm Ni^2+^, it is worth pointing out that the stimulatory effect of Ni^2+^ was lower with 25 mm [Na^+^]*_i_* than the one observed with 6 mm [Na^+^]*_i_* (Fig. 1*C*). This suggests that, although the inhibitory action of [Na^+^]*_i_* is smaller with Ni^2+^ than with Zn^2+^, it is not fully eliminated.

##### Differential Modulation of the Peak Current Amplitude by Ni^2+^ and Zn^2+^

**FIGURE 7.**
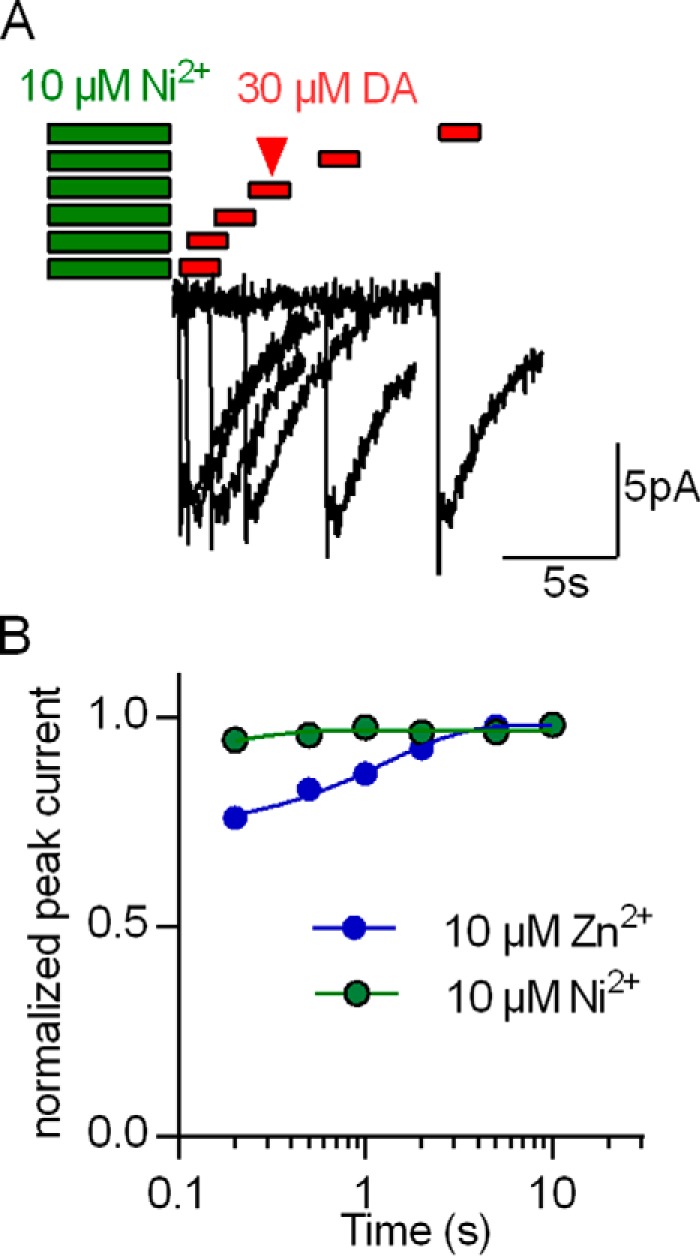
**Comparison of Ni^2+^- and Zn^2+^-induced changes in the peak current.** *A*, protocol for determining the effect of Ni^2+^ (or Zn^2+^) on the peak current by hDAT. A HEK293 cell stably expressing hDAT was clamped to −60 mV and exposed to 10 μm Ni^2+^ for 5 s. After wash periods of 0.2, 0.5, 1, 2, and 5 s, a peak current was triggered by rapid application of 30 μm dopamine (0.5 s). The original traces are from a representative experiment that was reproduced in seven independent experiments with 10 μm Ni^2+^ and Zn^2+^, respectively. *B*, recovery of the peak current following application of 10 μm Ni^2+^ (*green circle*s) and 10 μm Zn^2+^ (*blue circles*), respectively. The peak current amplitudes were normalized to the respective largest current and plotted as a function of the wash time interval. The two sets of data points were each fit to a monoexponential function (*green* and *blue lines).* The fits yielded the following parameters: Ni^2+^, T_recovery_ = 0.15 s (0.07–5.66), blocked fraction 13% (10–17); Zn^2+^, T_recovery_ = 1.25 s (0.82–2.5), blocked fraction 28% (22–33). Data represent mean and 95% confidence interval (in parentheses). The estimated blocked fractions were significantly different (*p* < 0.0001, F-test).

The substrate-induced peak current reflects the initiation of the transport cycle, where substrate and co-substrate ions are bound by the transporter and moved through the electric field of the membrane (18, 19). Zn^2+^ decreases the amplitude of this substrate-induced peak current (2), presumably because Zn^2+^ does not only favor the return of the empty transporter (forward transport mode) but also the return of the substrate-bound transporter to the outward-facing state (substrate exchange mode) (2). Accordingly, the charge that gives rise to the inwardly directed peak current is moved into the opposite direction, and, hence, the peak current amplitude is decreased. Ni^2+^ and Zn^2+^ differed in their ability to stimulate steady-state transport. In the micromolar range, Ni^2+^ uniformly stimulated substrate uptake, but Zn^2+^ gave rise to a stimulation at 1 μm and inhibition at 10 μm (*cf.* Fig. 5*A*). This suggests that Zn^2+^ and Ni^2+^ do not affect the conformational transitions hDAT must undergo during the transport cycle in the same way. Hence, we compared the effect of Ni^2+^ on the dopamine-induced peak current by employing the protocol shown in Fig. 7*A*. We applied 10 μm Ni^2+^ or Zn^2+^ for 5 s to a HEK293 cell expressing hDAT. Subsequently, test pulses of 30 μm dopamine were applied immediately or 0.2, 0.5, 1, and 2 s after removal of the metal ion. As can be seen in Fig. 7*B*, the effect of Ni^2+^ on the peak current was much smaller than that of Zn^2+^. Although Zn^2+^ decreased the peak current amplitude by about 30%, Ni^2+^ only affected it to a very minor extent.

##### Amphetamine-induced Substrate Release in the Presence of Zn^2+^ and Ni^2+^

**FIGURE 8.**
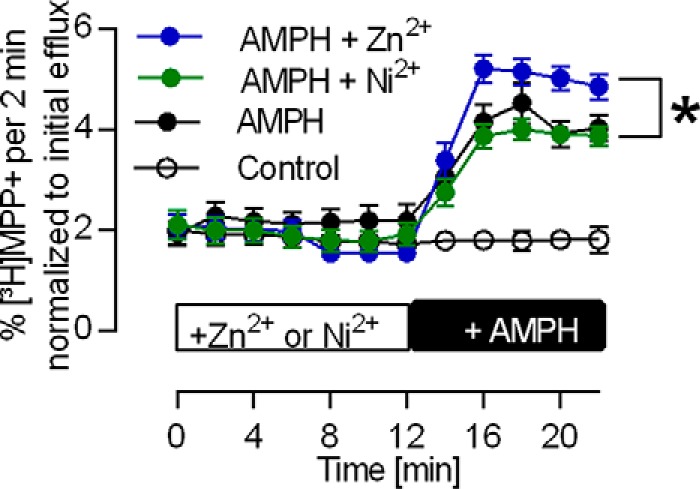
**Stimulation of amphetamine-induced [^3^H]MPP^+^ release by Zn^2+^ but not Ni^2+^.** HEK293 cells transfected with hDAT loaded with [^3^H]MPP+ were superfused with KHP buffer for 45 min (see “Experimental Procedures”). Thereafter (*time point 0*), 2-min fractions were collected. The prior interval of 45 min allowed for establishing a stable baseline as verified by the first seven fractions. After 12 min, 3 μm AMPH was added, which resulted in [^3^H]MPP+ release (*black circles*). This increase was larger in the presence of 10 μm Zn^2+^ (*blue circles*) but unchanged in the presence of 10 μm Ni^2+^ (*green circles*). The data are averages of 12 experiments. The data points at 20 and 22 min were used to conduct a one-way analysis of variance (Kruskal-Wallis test) followed by Dunn's multiple comparisons test (post hoc test). The difference between AMPH and AMPH + 10 μm Zn^2+^ is significant (*p* = 0.026).

During amphetamine-induced substrate release, DAT operates in the substrate exchange mode rather than the forward transport mode, *i.e.* amphetamine is carried inward and exchanged for dopamine (25, 26, 27). As outlined above, Zn^2+^ inhibits the peak current because it favors substrate exchange. Accordingly, Zn^2+^ increases amphetamine-induced substrate release mediated by DAT (21). In contrast, Ni^2+^ reduced the peak current amplitude only to a very modest extent (*cf.* Fig. 7) and did not block the steady-state current (*i.e.* the forward transport mode) in the presence of elevated [Na^+^]*_i_*. Based on our model, these findings predict that, contrary to Zn^2+^, Ni^2+^ does not enhance amphetamine-induced substrate efflux. This conjecture was verified (Fig. 8). In hDAT-expressing HEK293 cells that had been preloaded with [^3^H]MPP^+^, superfusion with 3 μm d-amphetamine (AMPH) caused a robust release of radioactive substrate that was further augmented by the concomitant application of 10 μm Zn^2+^. In contrast, Ni^2+^ did not enhance amphetamine-induced substrate release.

## Discussion

DAT has a tetrahedral Zn^2+^-binding site. The coordination sphere is comprised of H193 in extracellular loop 2 (EL2), His^375^ and Glu^396^, which reside on top of transmembrane helix 7 in the first helical part of extracellular loop 4 (EL4A) and on top of transmembrane helix 8 in the second helix of extracellular loop 4 (EL4B), respectively (28), and Asp^206^ in EL2 (29). The extracellular loops are thought to be flexible, in particular the extended EL2 (29). Accordingly, it is not surprising that this binding site can accommodate transition metals other than Zn^2+^ even when their optimum coordination geometries differ (5). In fact, our observations are in line with the general prediction of the Irving-William series. It was, however, surprising that the effects elicited by individual metal ions, in particular Zn^2+^, Ni^2+^, and Cu^2+^, differed substantially. Ni^2+^ and Cu^2+^ uniformly stimulated and inhibited, respectively, the forward transport mode of DAT. In contrast, Zn^2+^ elicited biphasic effects. In addition, and most importantly, Zn^2+^ was the only transition metal ion that also promoted the substrate exchange mode. The different modes of action of the metals must be accounted for by a plausible model of the transport cycle. It is *a priori* reasonable to posit that occupancy of the Zn^2+^-binding site produces the same primary effect. In fact, this was the case. All effective metals shifted the EC_50_ of dopamine for eliciting the substrate-induced steady-state current, albeit to a different extent. The shift was most pronounced for Cu^2+^, followed by Ni^2+^ and Zn^2+^ (and Co^2+^). These shifts indicate that Zn^2+^, and, to a larger extent, Ni^2+^ and Cu^2+^, destabilized the substrate-bound conformation(s). Destabilization is most parsimoniously explained by preferred binding of these metals to the apo conformation(s) of DAT. Thermodynamics dictate that the effect on affinity must be reciprocal. If the transition metal lowers the affinity of DAT for substrate, then raising the substrate concentration must lower the affinity for metals. In addition, because individual metals differed in their ability to shift the EC_50_ of dopamine, they must discriminate between apo and substrate-bound transporters to different extents, with Zn^2+^ and Cu^2+^ having the least and most pronounced affinity differences, respectively.

**FIGURE 9.**
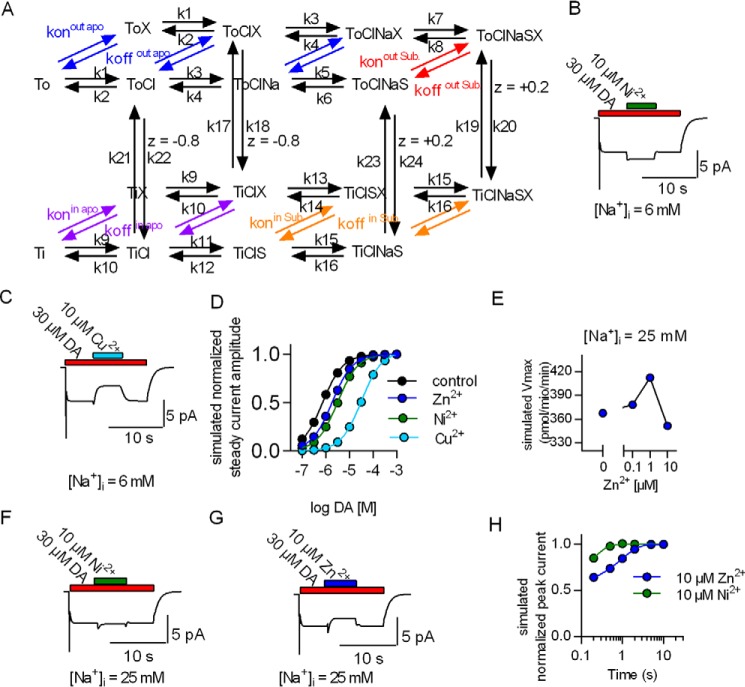
**A kinetic model of the action of first-row transition metals on the transport cycle of DAT.** *A*, the model scheme, which is the same as in Li *et al.* (2). *X* indicates transition metals. For each metal there is a separate set of parameters (see supplemental Table 1). Parameters are shared between these sets when they describe conformational rearrangements and binding reactions within DAT that are unaffected by the metals. *Red* and *orange arrows* indicate metal binding to substrate-bound states, and *arrows* in *violet* and *blue* indicate metal binding to apo states. *B* and *C*, simulated current evoked by 30 μm dopamine stimulated by 10 μm Ni^2+^ (*B*) or inhibited by 10 μm Cu^2+^ (*C*). *D*, simulated concentration dependence of dopamine-induced currents in the absence and presence of 10 μm Zn^2+^, Ni^2+^, and Cu^2+^, respectively. *E*, simulated *V*_max_ of dopamine uptake by DAT in the absence and presence of 1 and 10 μm Zn^2+^, assuming 25 mm [Na^+^]*_i_. F*, simulated currents induced by 30 μm dopamine with [Na^+^]*_i_* set to 25 mm. Under this condition, the model predicts current stimulation by Ni^2+^ and inhibition by Zn^2+^. *G*, simulated peak current recovery after application of 10 μm Ni^2+^ (*green circles*) or Zn^2+^ (*blue circles*). The simulations evidently account for the measured data.

We implemented this information into a kinetic model (18) that was extended to account for metal-bound states (Fig. 9*A*). This allowed for recapitulating all experimental findings. 1) It is, for instance, possible to account for the observation that low Zn^2+^ concentrations (1 μm) increased substrate uptake in the presence of high [Na^+^]*_i_* and for the biphasic effect of Zn^2+^ on substrate uptake. The conformation that displays the highest affinity for Zn^2+^ is the outward-facing apo state of DAT; the Zn^2+^-binding site is fully occupied at 1 μm. Upon substrate binding, the affinity at the binding site decreases, and Zn^2+^ dissociates. Thus, at low concentrations (1 μm), Zn^2+^ interacts almost exclusively with the apo conformations. This leads to an acceleration of the rate-limiting step in the transport cycle and stimulation of substrate uptake. At elevated concentrations (*e.g.* 10 μm), Zn^2+^ also binds to the substrate-loaded conformations. This gives rise to Zn^2+^-induced inhibition when [Na^+^]*_i_* is high (Fig. 9, *E* and *G*). 2) The key actions of Ni^2+^ were reproduced when the observation that Ni^2+^ induced a larger shift in the EC_50_ of dopamine than Zn^2+^ was incorporated into the model. These actions are stimulation of the dopamine-induced steady-state current by Ni^2+^ (Fig. 9*B*), the modest effect of Ni^2+^ on the peak current (Fig. 9*H*), and the stimulatory action of Ni^2+^ despite elevated [Na^+^]*_i_* (Fig. 9*F*). 3) Finally, the model also accounts for current inhibition by Cu^2+^ (Fig. 9*C*). In the presence of Cu^2+^, the shift in EC_50_ was most pronounced, *i.e.* larger than with any other transition metal (*cf.* Figs. 3*C* and 9*D*). This observation indicated that Cu^2+^ had the highest preference for the apo conformations. Hence, its uniform inhibitory action can be rationalized as follows. In the absence of Cu^2+^, 30 μm dopamine is a saturating concentration that induces maximal currents and allows the transporter to cycle at maximum velocity in the forward transport mode. In contrast, Cu^2+^ lowers the affinity of dopamine to an extent that a dopamine concentration of 30 μm does not suffice for saturation (Fig. 9*D*). This leads to a decrease in current amplitude, which reflects inhibition of dopamine transport by Cu^2+^. Thus, the parsimonious explanation for the action of transition metals on DAT is the reciprocal effect of substrate and metal: binding of one lowers the affinity for the respective other. Given the location of the coordinating residues, it is attractive to assume that occupancy of the Zn^2+^-binding site by metals precludes the transition of DAT from the outward open to the occluded state. By definition, this reduces the apparent substrate affinity because the substrate can more readily dissociate from the outward open than from the occluded state. Conversely, in the occluded state, the relative position of the residues that coordinate the transition metal is likely to change. This is predicted to translate into a decline in affinity for the metal.

The affinity of Mn^2+^ for the Zn^2+^-binding site of DAT was so low that there was no appreciable occupancy up to 100 μm Mn^2+^. This low affinity is consistent with the Irving-Williams series (5, 8, 9). Based on this low affinity, it appears unlikely that mismetallation of the Zn^2+^-binding site by manganese is relevant to the mechanistic understanding of manganese-induced dopamine deficiency (10–15). In contrast, in the low micromolar range, Cu^2+^ caused a profound inhibition of DAT. Hence, this inhibition may be relevant to understand symptoms that occur in the course of Wilson's disease for the following reasons. 1) Although it is difficult to estimate the free concentration of Cu^2+^ in the brain, the serum concentration of free Cu^2+^ (*i.e.* not bound to coeruloplasmin) is in the micromolar range (30). The concentration of Cu^2+^ at the site of dopaminergic projections is thought to exceed that in serum because Cu^2+^ accumulates in several brain regions, including the corpus striatum. Thus, Cu^2+^ levels in the brain are likely to reach concentrations that impair retrieval of dopamine by DAT and thus lead to a hypodopaminergic state. 2) Imaging studies suggest that a presynaptic dopaminergic deficit contributes to the neurological symptoms from which patients with Wilson's disease suffer (31–33). 3) In addition, patients with Wilson's disease are very susceptible to side effects of neuroleptic drugs that arise from dopamine receptor blockage in the caudate nucleus and putamen. When erroneously treated with both typical and atypical neuroleptic/antipsychotic drugs, these patients are prone to develop drug-induced extrapyramidal symptoms (34). This enhanced susceptibility presumably arises from low D_2_ receptor occupancy by endogenous dopamine because of impaired synaptic release. 4) Finally, administration of zinc (*e.g.* 150 mg of zinc acetate/day) is an effective treatment for Wilson's disease and can be used as an alternative to chelation therapy with d-penicillamine or trientine (16, 30). The effect relies on the Zn^2+^-dependent induction of metallothionein into the gut epithelium and the resulting reduced systemic bioavailability of dietary copper (16). Interestingly, Zn^2+^ has been found to be particularly effective in improving neurological deficits (30, 35) even when prior chelation therapy failed (36, 37). Based on this circumstantial evidence and on our observations, we propose that displacement of Cu^2+^ from the Zn^2+^-binding site of DAT may contribute to the therapeutic efficacy of Zn^2+^ in Wilson's disease.

Occupancy of the allosteric Zn^2+^-binding site allows for a wide range of effects, *i.e.* stimulation and inhibition of the forward transport mode and enhancing the substrate exchange mode. It is likely that this binding site can be addressed by compounds other than transition metals. Allosteric activators of the forward transport mode are of particular interest. There are several loss-of-function mutations in the coding sequence of human DAT that lead to a syndrome of infantile or juvenile dystonia and parkinsonism (14). The vast majority of these mutations result in defective folding and, hence, retention of the mutated proteins in the endoplasmic reticulum. However, there are some mutants that do reach the cell surface but fail to transport dopamine with adequate turnover rates. A search for allosteric activators is therefore justified because they may act as correctors and restore the forward transport mode to a velocity that is commensurate with normal dopaminergic transmission (40). In fact, it appears worthwhile to test whether Ni^2+^ (or any other transition metal) accelerates transport in these mutants.

## Experimental Procedures

### 

#### 

##### Whole-cell Patch Clamp

HEK293 cells stably expressing hDAT or transiently expressing hDAT H193K were seeded at low density 24 h before recordings. Currents by the transporter were measured in the whole-cell patch clamp configuration. The electrode resistances were between 2–5 megohms. For the majority of the recordings, we used the following internal solution: 133 mm potassium gluconate, 6 mm NaCl, 1 mm CaCl_2_, 0.7 mm MgCl_2_, 10 mm EGTA, and 10 mm HEPES adjusted to pH 7.2 with KOH. For some experiments we used an internal solution containing 25 mm Na^+^, 25 mm NaCl, 125 mm KCl, 1 mm CaCl_2_, 0.7 mm MgCl_2_, 10 mm EGTA, and 10 mm HEPES (pH 7.2 with KOH). The external solution in all experiments was 140 mm NaCl, 3 mm KCl, 2.5 mm CaCl_2_, 2 mm MgCl_2_, 20 mm glucose, and 10 mm HEPES adjusted to pH 7.4 with NaOH. For rapid solution exchange, we used an OctaFlow superfusion device (ALA Scientific, Farmingdale, NY). Cells were continuously superfused either with blank external solution or an external solution containing dopamine, respectively. For the solutions containing Mn^2+^, Co^2+^, Ni^2+^, Zn^2+^, or Cu^2+^, we used the respective chloride salts (Sigma-Aldrich, St. Louis, MO). For current acquisition, we employed an Axopatch 200B amplifier and pClamp 10.2 software (Molecular Devices, Sunnyvale, CA). The cells were clamped to −60 mV, and the washout period following substrate application was 30 s in all cases. Current traces were filtered at 1 kHz and digitized at 10 kHz using a Digidata 1550 (Molecular Devices). The currents induced by dopamine were quantified using Clampfit 10.2 software. Passive holding currents were subtracted, and the traces were filtered using a 100-Hz digital Gaussian low-pass filter.

##### Uptake Assay

HEK293 cells stably expressing hDAT were seeded on 48-well plates precoated with poly-d-lysine (0.5 × 10^5^ cells/well) 24 h before the assay. Cells were first washed with 500 μl/well Krebs-HEPES buffer (KHP): 130 mm NaCl, 10 mm HEPES, 1.3 mm KH_2_PO_4_, 1.5 mm CaCl_2_, and 0.5 mm MgSO_4_ (pH 7.4) with NaOH. The cells were then incubated in 200 μl of KHP buffer containing 0.1 μm [^3^H]dopamine and diluted with the respective cold substrates to reach final concentrations ranging from 0.1–100 μm, both in the absence and presence of 1 and 10 μm ZnCl_2_ or NiCl_2,_ respectively. The cells were incubated at room temperature for 1 min with [^3^H]dopamine. A subsequent wash with 500 μl/well of ice-cold KHP buffer terminated uptake. Cells were finally lysed with 500 μl of 1% SDS, transferred into 2 ml of scintillation mixture (Rotiszint Eco Plus LSC, Art. 0016.3, Carl Roth GmbH + Co. KG, Karlsruhe, Germany), and counted in a Packard 2300TR TriCarb liquid scintillation analyzer (PerkinElmer Life Sciences).

##### Superfusion Experiments

HEK293 cells stably expressing hDAT (2.5 × 10^4^ cells/coverslip) were preloaded with 0.2 μm [^3^H]MPP^+^ for 30 min. The coverslips were transferred to superfusion chambers and perfused with KHP buffer (0.7 ml/min at 22 °C). After an initial wash period of 45 min, fractions were collected every 2 min. The collection of the first fraction defined time point zero. Cells were exposed 10 μm ZnCl_2_ or 10 μm NiCl_2_ after two baseline fractions (4 min). 3 μm d-amphetamine was added to the superfusion buffer after 12 min. All fractions were analyzed by liquid scintillation counting (see above).

##### Modeling

The recorded currents were emulated with a previously published kinetic model of the transport cycle of DAT (2). This model was extended to account for the binding of Zn^2+^, Ni^2+^, and Cu^2+^. The time-dependent changes in state occupancies were evaluated by numerical integration of the resulting system of differential equations using Systems Biology Toolbox (38) and Matlab 2012a (Mathworks, Natick, MA). The voltage dependence of individual rates was modeled according to Laeuger (39), assuming a symmetric barrier as k_ij_ = k0_ij_exp(−zQ_ij_FV / 2RT), with F = 96,485 Cmol^−1^, *r* = 8.314 JK^−1^mol^−1^, V the membrane voltage in volts, and T = 293 K. The extra- and intracellular ion concentrations were set to the values used in the experiments. Substrate uptake was modeled as (TiClS × kSi_off_ − TiCl × Si × kSi_on_ + TiClSZn × kSi_off_ − TiClZn × Si × kSi_on_) × NC/NA, where NC is the number of transporters and NA is the Avogadro constant. Currents and substrate uptake were simulated assuming a transporter density of 25 × 10^6^/cell.

## Author Contributions

Y. L., F. P. M., P. S. H., K. S., M. F., and W. S. conceptualized the study and designed the experiments. P. S. H., K. S., and W. S. designed the model. Y. L., F. P. M., and V. B. performed all experiments and analyzed the data. Y. L., F. P. M., H. H. S., M. F., and W. S. wrote the manuscript.

## Supplementary Material

Supplemental Data

## Footnotes

3 The abbreviations used are:

DAT: dopamine transporter
hDAT: human dopamine transporter
AMPH: d-amphetamine
EL: extracellular loop
DA: dopamine
EC_50_: half-maximal effective concentration.
