# Supplementary material for: Occupancy of the Zinc-binding Site by Transition Metals Decreases the Substrate Affinity of the Human Dopamine Transporter by an Allosteric Mechanism
Source: J Biol Chem. 2017 Jan 17;292(10):4235–43. doi: 10.1074/jbc.M116.760140 (PMC5354487; doi:10.1074/jbc.M116.760140)
Supplement: Supplemental Data [file supp_292_10_4235__index.html]

Occupancy of the Zinc Binding-site by Transition Metals decreases the Substrate Affinity of the Human Dopamine Transporter by an Allosteric Mechanism. — Occupancy of the Zinc-binding Site by Transition Metals Decreases the Substrate Affinity of the Human Dopamine Transporter by an Allosteric Mechanism — Allosteric Modulation of the Transport Cycle of DAT — Supplemental Data 

# Occupancy of the Zinc-binding Site by Transition Metals Decreases the Substrate Affinity of the Human Dopamine Transporter by an Allosteric Mechanism

## Supplemental Data

- Supplemental Table (.docx, 17 KB) - Table
