# Supplementary material for: Occupancy of the Zinc-binding Site by Transition Metals Decreases the Substrate Affinity of the Human Dopamine Transporter by an Allosteric Mechanism
Source: J Biol Chem. 2017 Jan 17;292(10):4235–43. doi: 10.1074/jbc.M116.760140 (PMC5354487; doi:10.1074/jbc.M116.760140)
Supplement: Supplemental Data [file 10.1074_M116.760140_jbc.M116.760140-1.docx]

| model rates | Zn^2+^ | Ni^2+^ | Cu^2+^ |
| --- | --- | --- | --- |
| k1: To(X)🡺ToCl(X) | 5*10^5^ s^-1^*mol^-1^ | 5*10^5^ s^-1^*mol^-1^ | 5*10^5^ s^-1^*mol^-1^ |
| k2: ToCl(X)🡺To(X) | 2*10^3^ s^-1^ | 2*10^3^ s^-1^ | 2*10^3^ s^-1^ |
| k3: ToCl(X)🡺ToClNa(X) | 3*10^5^ s^-1^*mol^-1^ | 3*10^5^ s^-1^*mol^-1^ | 3*10^5^ s^-1^*mol^-1^ |
| k4: ToClNa(X)🡺ToCl(X) | 1*10^4^ s^-1^ | 1*10^4^ s^-1^ | 1*10^4^ s^-1^ |
| k5: ToClNa🡺ToClNaS | 2*10^6^ s^-1^*mol^-1^ | 2*10^6^ s^-1^*mol^-1^ | 2*10^6^ s^-1^*mol^-1^ |
| k6: ToClNaS🡺ToClNa | 10 s^-1^ | 10 s^-1^ | 10 s^-1^ |
| k7: ToClNaX🡺ToClNaSX | 2*10^6^ s^-1^*mol^-1^ | 2*10^6^ s^-1^*mol^-1^ | 2*10^6^ s^-1^*mol^-1^ |
| k8: ToClNaSX🡺ToClNaX | 100 s^-1^ | 1*10^3^ s^-1^ | 1*10^4^ s^-1^ |
| k9: Ti(X)🡺 TiCl(X) | 1*10^5^ s^-1^*mol^-1^ | 1*10^5^ s^-1^*mol^-1^ | 1*10^5^ s^-1^*mol^-1^ |
| k10: TiCl(X)🡺 Ti(X) | 2*10^3^ s^-1^ | 2*10^3^ s^-1^ | 2*10^3^ s^-1^ |
| k11: TiCl🡺 TiClS | 2*10^6^ s^-1^*mol^-1^ | 2*10^6^ s^-1^*mol^-1^ | 2*10^6^ s^-1^*mol^-1^ |
| k12: TiClS🡺 TiCl | 10 s^-1^ | 10 s^-1^ | 10 s^-1^ |
| k13: TiClX🡺TiClSX | 2*10^5^ s^-1^*mol^-1^ | 2*10^5^ s^-1^*mol^-1^ | 2*10^5^ s^-1^*mol^-1^ |
| k14: TiClSX🡺TiClX | 10 s^-1^ | 100 s^-1^ | 1*10^3^ s^-1^ |
| k15: TiClNaS(X)🡺TiClNa(X) | 3*10^5^ s^-1^*mol^-1^ | 3*10^5^ s^-1^*mol^-1^ | 3*10^5^ s^-1^*mol^-1^ |
| k16: TiClS(X)🡺 TiClNaS(X) | 1*10^4^ s^-1^ | 1*10^4^ s^-1^ | 1*10^4^ s^-1^ |
| k17: TiClX🡺 ToClX | 1 s^-1^ | 1 s^-1^ | 1 s^-1^ |
| k18: ToClX🡺 TiClX | 0.1 s^-1^ | 0.01 s^-1^ | 0.01 s^-1^ |
| k19: TiClNaSX🡺 ToClNaSX | 1*10^3^ s^-1^ | 1*10^4^ s^-1^ | 1*10^4^ s^-1^ |
| k20: ToClNaSX🡺 TiClNaSX | 100 s^-1^ | 100 s^-1^ | 100 s^-1^ |
| k21: TiCl🡺 ToCl | 0.6 s^-1^ | 0.6 s^-1^ | 0.6 s^-1^ |
| k22: ToCl🡺 TiCl | 0.6 s^-1^ | 0.6 s^-1^ | 0.6 s^-1^ |
| k23: TiClNaS🡺 ToClNaS | 100 s^-1^ | 100 s^-1^ | 100 s^-1^ |
| k24: ToClNaS🡺 TiClNaS | 100 s^-1^ | 100 s^-1^ | 100 s^-1^ |
| Zn^2+^k_on_ out apo | 1*10^7^ s^-1^*mol^-1^ | 1*10^7^ s^-1^*mol^-1^ | 1*10^7^ s^-1^*mol^-1^ |
| Zn^2+^k_off_ out apo | 1 s^-1^ | 5 s^-1^ | 0.3 s^-1^ |
| Zn^2+^k_on_ out Sub | 1*10^7^ s^-1^*mol^-1^ | 1*10^7^ s^-1^*mol^-1^ | 1*10^7^ s^-1^*mol^-1^ |
| Zn^2+^k_off_ out Sub | 10 s^-1^ | 500 s^-1^ | 300 s^-1^ |
| Zn^2+^k_on_ in apo | 1*10^7^ s^-1^*mol^-1^ | 1*10^7^ s^-1^*mol^-1^ | 1*10^7^ s^-1^*mol^-1^ |
| Zn^2+^k_off_ in apo | 10 s^-1^ | 50 s^-1^ | 30 s^-1^ |
| Zn^2+^k_on_ in Sub | 1*10^7^ s^-1^*mol^-1^ | 1*10^7^ s^-1^*mol^-1^ | 1*10^7^ s^-1^*mol^-1^ |
| Zn^2+^k_off_ in Sub | 100 s^-1^ | 5*10^4^ s^-1^ | 3*10^5^ s^-1^ |

Supplemental Table: Shown are the rates used to model the data in figure 9 (main manuscript). Highlighted in yellow are those rates that were adopted to account for the differences in the respective metal actions.
